# Profiling the Biological Characteristics and Transitions through Upper Tract Tumor Origin, Bladder Recurrence, and Muscle-Invasive Bladder Progression in Upper Tract Urothelial Carcinoma

**DOI:** 10.3390/ijms23095154

**Published:** 2022-05-05

**Authors:** Keisuke Shigeta, Kazuhiro Matsumoto, Nobuyuki Tanaka, Shuji Mikami, Takeo Kosaka, Yota Yasumizu, Toshikazu Takeda, Ryuichi Mizuno, Eiji Kikuchi, Mototsugu Oya

**Affiliations:** 1 Department of Urology, Keio University School of Medicine, Tokyo 160-8582, Japan; keisukeshigeta@yahoo.co.jp (K.S.); urotanaka@gmail.com (N.T.); takemduro@gmail.com (T.K.); y.yasumizu0707@gmail.com (Y.Y.); ttakeda8156@yahoo.co.jp (T.T.); moya3563@yahoo.co.jp (R.M.); moto-oya@z3.keio.jp (M.O.); 2 Division of Diagnostic Pathology, Keio University School of Medicine, Tokyo 160-8582, Japan; mikamishuji@gmail.com; 3 Department of Urology, St. Marianna University School of Medicine, Kanagawa 216-8511, Japan; eiji-k@kb3.so-net.ne.jp

**Keywords:** fibroblast growth factor receptor 3, intravesical recurrence, molecular characteristic, muscle-invasive bladder carcinoma, upper tract urothelial carcinoma

## Abstract

To evaluate biological characteristics and transitions of upper tract urothelial carcinoma (UTUC) through metachronous bladder tumors after radical nephroureterectomy (RNU), we conducted immunohistochemical (IHC) staining of tumor specimens of UTUC tumor origin, non-muscle-invasive bladder cancer (NMIBC) and MIBC progressed after intravesical recurrence (IVR), and bladder primary MIBC. Fibroblast growth factor receptor 3 (FGFR3), p53, cytokeratin 5/6 (CK5/6), and CK20 were stained to examine expression rates. After expression assessment with heatmap clustering, the overexpression of four biomarkers from UTUC origin to metachronous MIBC progression was analyzed with clinicopathological variables. We found that high CK20 and low CK5/6 expression were both observed in UTUC tumor origin and subsequent NMIBC after RNU. By investigating molecular expression in the IVR specimen, we observed that low pT stage bladder recurrence occupied the majority of CK20 high CK5/6 low expression, but would change to CK20 low CK5/6 high expression as it progressed to MIBC. UTUC metachronous MIBC has different characteristics compared with bladder primary MIBC, which comprises favorable biological features such as high FGFR3 expression, and follows favorable prognosis compared to those without FGFR3 expression. The present study demonstrated that the biological characteristics of UTUC tumor origin shifts from luminal to basal-like features with progression to MIBC, but FGFR3 expression taken over from UTUC origin may comprise a favorable entity compared to primary MIBC.

## 1. Introduction

Upper tract urothelial carcinoma (UTUC) is relatively uncommon, accounting for approximately 5% of urothelial malignancies [1]. A major cause of concern for UTUC patients is intravesical recurrence (IVR) after radical nephroureterectomy (RNU), the incidence of which is reported to be approximately 15–50% [2]. Although the majority of IVR pathologically results in pTa or pT1 with additional transurethral resection (TUR), some bladder cancers originating from UTUC develop as muscle-invasive bladder cancer (MIBC) at the initial bladder tumor recurrence or progression to secondary MIBC after repeated IVR development [3].

With recent advances in genomic analysis, the mutational landscapes of primary UTUC and primary bladder urothelial carcinoma (BUC) are now considered to have slightly different molecular aspects. Audenet and Sfakianos et al. reported that fibroblast growth factor receptor 3 (FGFR3) and HRAS were more significantly altered in UTUC, whereas TP53, RB1, and ERBB2 were more often altered in BUC, which supports the molecular differences between UTUC and BUC [4,5]. Robinson et al. also indicated that UTUC is enriched with tumors belonging to the luminal papillary subtype characterized by FGFR3 gene expression signatures, which are associated with favorable outcomes in UC patients [6]. However, the genomic and/or molecular biology regarding UTUC tumor origin and bladder cancer which recurred after RNU are under investigation. A recent transcriptome analysis performed by Petros et al. revealed that UTUC metachronous bladder tumors largely maintain the molecular membership of the initial tumor, which suggests the predominance of the intraluminal seeding theory for bladder recurrence in UTUC patients [7]. However, standard subtyping based on transcriptomic analysis is relatively expensive. Thus, there is a lack of evidence on how molecular characteristics of the UTUC tumor origin would transit or change in the process of bladder tumor recurrence and bladder tumor progression in real-world clinical settings.

Since the Cancer Genome Atlas Network described molecular subtypes such as basal and luminal subtypes in 2014, several molecular markers such as cytokeratin (CK)5/6, CK14, CK20, and GATA3 have been introduced for immunohistochemical (IHC) staining for simple molecular subclassification [8]. In particular, CK5/6 and CK20 are often used in clinicopathological examinations, as they have been shown to have a correlation with diagnostic and prognostic implications in several studies [9,10]. However, these molecular markers are mainly utilized for bladder primary UC, and therefore, it remains unclear whether molecular classification can be also applied for UTUC tumor origin, bladder tumor after IVR development, and bladder tumor that progresses to secondary MIBC after repeated IVR development.

Based on these scenarios, the biology of UTUC tumor origin to metachronous MIBC progression has not yet been clearly investigated. Since UTUC and IVR tumor specimens are developed from identical organisms that allow unique opportunities to investigate temporal and anatomical disease biology, we aimed to clarify the biological pattern and overtime transition among UTUC, IVR, and UTUC metachronous MIBC. Moreover, we also aimed to identify the biological differences between UTUC-derived and bladder primary MIBC.

## 2. Results

### 2.1. The Molecular Biomarker Patterns in 214 UTUC Tumor Samples

**Figure 1:**
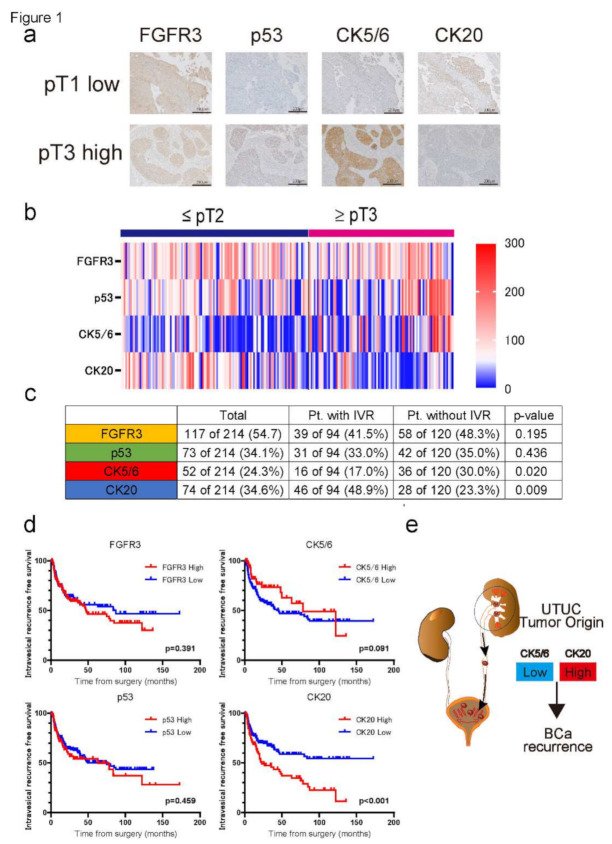
(**a**) Representative immunohistochemistry (IHC) staining of FGFR3, p53, CK5/6, and CK20 in surgical specimens; pTa low-grade upper tract urothelial carcinoma (UTUC) patients (upper panel) and pT3 high-grade UTUC patients (lower panel). The field scale bar = 200 µm. (**b**) Heatmap describing the IHC score of FGFR3, p53, CK5/6, and CK20 in UTUC patients (*n* = 214). Heatmap is divided by pT2 or low (*n* = 116) and pT3 or high (*n* = 98). The histoscore (H-score) was calculated by applying the following formula: mean percentage × intensity (range 0–300). (**c**) Table describing the rate of high expression group in each molecular biomarker. Expression rates were compared between patients who developed intravesical recurrence (IVR) and who did not develop IVR after radical nephroureterectomy (RNU). Chi-squared test was used to assess the significance of differences. (**d**) K–M curves comparing the IVR free survival rates in UTUC patients compared by low versus high FGFR3 expression, low versus high p53 expression, low versus high CK5/6 expression, and low versus high CK20 expression. Log-rank test was used to assess the significance of differences. (**e**) A schema describing the molecular characteristics of UTUC that are likely to develop IVR. As the multivariate analysis revealed, CK20 high and CK5/6 low expression tumor specimens were the independent risk factors for developing IVR after radical nephroureterectomy.

Among the 214 UTUC patients, the median age was 73 (47–92) and the median follow-up duration was 58.5 (1.8–172.5) months. The clinicopathological characteristics of patients with UTUC who underwent RNU are shown in Table 1. Overall, 94 (43.9%) patients developed subsequent IVR during follow-up. Lower pT stage, higher tumor grade, and lower LVI rate were observed in the IVR group than in the non-developed group. Referring to the molecular markers, FGFR3, p53, CK5/6, and CK20 were stained using a standard protocol. Representative IHC staining for all molecular markers is shown in Figure 1a. The mean H-scores were FGFR3: 98.9 ± 51.4, p53: 84.6 ± 61.7, CK5/6: 58.3 ± 65.6, and CK20: 56.3 ± 44.1. According to the heatmap shown in Figure 1b, the H-score of CK5/6 tended to show a high expression in the advanced pT stage (pT3 or higher: 81.4 ± 79.3 vs. pT2 or lower: 38.9 ± 42.9), while CK20 showed a lower expression in the advanced tumor stage compared to its counterparts (pT2 or lower: 67.8 ± 52.4 vs. pT3 or higher: 37.2 ± 43.3). Contrarily, the H-score of FGFR3 and p53 expression did not show significant differences between both the early and advanced T stage (99.1 ± 51.1 vs. 98.8 ± 52.3 and 80.7 ± 73.9 vs. 80.3 ± 49.1, respectively). Based on the H-scores obtained from the four molecular markers, we set cut-off values defining high and low expression according to the ROC curve analysis (Supplemental Figure S1).

When focusing on patients who developed IVR, higher CK20 expression (*p* = 0.009) and lower CK5/6 expression (*p* = 0.020) were observed in UTUC tumor specimens that developed IVR (Figure 1c). There were no significant differences in FGFR3 and p53 expression in the UTUC tumor specimens. The Kaplan–Meier (K–M) analysis also revealed that the high CK20 expression group showed a lower bladder recurrence-free survival rate than its counterparts (*p* < 0.001), and a similar tendency was observed in the low CK5/6 expression group (*p* = 0.091). Multivariate analysis revealed that high CK20 (HR = 3.31, *p* < 0.001) and low CK5/6 (HR = 0.26, *p* < 0.001) expression in UTUC specimens were both independent factors for developing IVR as well as pT2 or lower T stage (HR = 1.94, *p* = 0.007) and a high tumor grade (HR = 1.61, *p* = 0.032; Table 2).

From the above results, we suspected that UTUC tumors with high CK20 and low CK5/6 expression, which are known as luminal-like subtypes, are likely to develop subsequent IVR after RNU.

### 2.2. Luminal–Basal Shift in IVR Specimens with the Progression of Muscle Invasion

**Figure 2:**
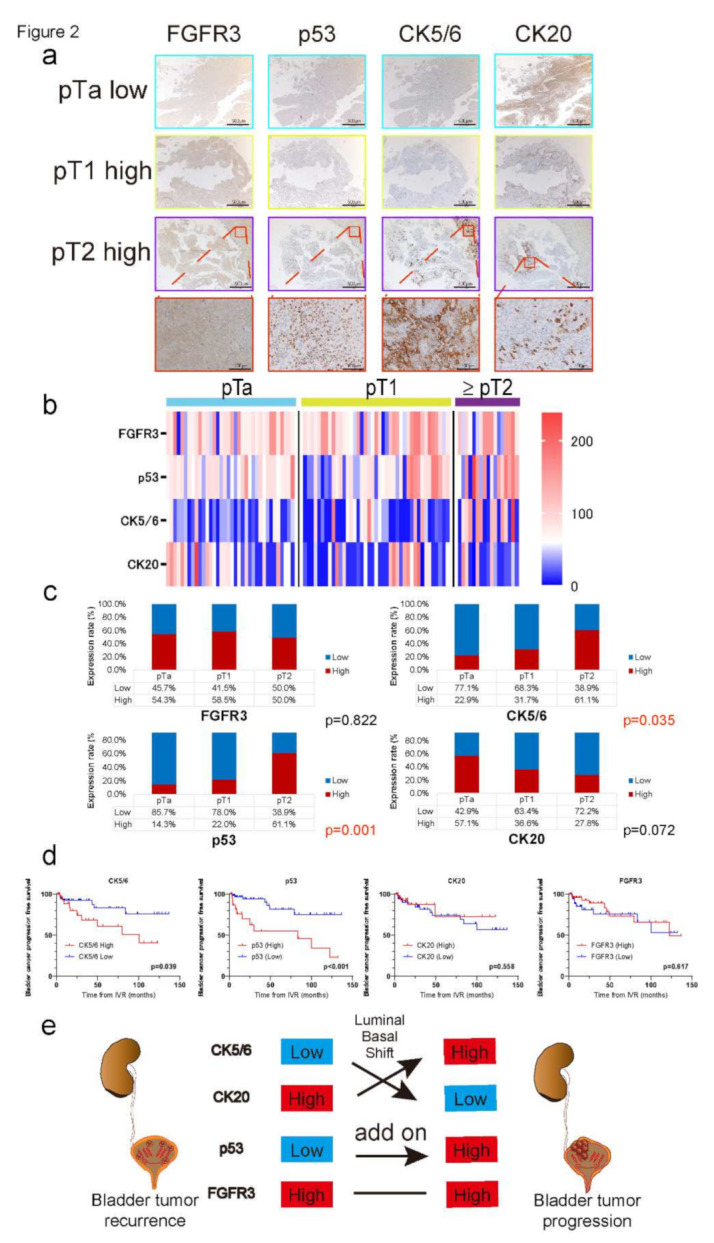
(**a**) Representative IHC staining of FGFR3, p53, CK5/6, and CK20 in surgical specimens after transurethral resection of bladder tumor (TURBT); pTa low-grade IVR specimen, pT1 high-grade IVR specimen, and pT2 high-grade IVR specimen are shown, respectively. Low-power field scale bar = 500 µm, and high-power field scale bar = 100 µm. (**b**) Heatmap describing the IHC score of FGFR3, p53, CK5/6, and CK20 in transurethral resection (TUR) specimens after IVR (*n* = 94). Heatmap is divided by pTa (*n* = 35), pT1 (*n* = 41), and pT2 or higher (*n* = 18). The H-score was calculated by applying the following formula: mean percentage × intensity (range 0–270). (**c**) Graph showing the transition of four molecular markers’ expressions classified with pathological T stage. P53 and CK5/6 showed significantly higher expressions at muscle-invasive stage (pT2) than that of non-muscle-invasive stage (pTa and/or pT1; *p* = 0.001, *p* = 0.035), while CK20 expression decreased as the tumor stage progressed to MIBC (*p* = 0.072). No significant differences were observed in FGFR3 expression along with tumor stage progression (*p* = 0.822). (**d**) K–M curves comparing the bladder cancer progression free survival rates in UTUC patients who developed IVR compared by low versus high FGFR3 expression, low versus high p53 expression, low versus high CK5/6 expression, and low versus high CK20 expression. Log-rank test was used to assess the significance of differences. (**e**) A schema describing the transition of molecular characteristics of IVR specimen. At the time of bladder tumor recurrence, CK20 high and CK5/6 low expressions (luminal-like) occupy the majority of IVR specimen. In the process of MIBC progression, however, the high CK20 expression rate decreased as it progressed to muscle invasive, whereas the high CK5/6 expression rate increased (basal-like). Schema suggests the concept of “luminal–basal shift” with p53 addition from low stage bladder tumor recurrence to high stage bladder tumor progression.

We further investigated the IHC staining of bladder tumor specimens obtained by TURBT after IVR development. The clinicopathological characteristics of patients who underwent TURBT after IVR development are shown in Table 3. The median follow-up time was 23.7 (3.8–122.4) months. Among the 94 patients who developed IVR, 37 (39.4%) had pTa, 39 (41.5%) had pT1, and 18 (19.1%) subsequently progressed to muscle invasion status. Of the four biomarkers, 52 (55.3%) showed high FGFR3, 25 (26.6%) showed high p53, 32 (34.0%) showed high CK5/6, and 40 (42.6%) showed high CK20 expression. Representative IHC staining results are shown in Figure 2a. By classifying the IVR specimens, we found that p53 expression gradually increased as it progressed to MIBC status, and CK20 high and CK5/6 low expression gradually switched to CK20 low and CK5/6 high as the pT stage increased from Ta to T2 or higher. The degrees of expression of the four molecular markers are shown in Figure 2b,c. Specifically, only 16.2% showed high p53 expression at the pTa stage but this significantly increased to 61.1% at ≥pT2 (*p* < 0.001). Likewise, high CK5/6 expression only accounted for 22.9% in pTa, but 61.1% showed high CK5/6 expression in the ≥pT2 stage, which was statistically significant (*p* = 0.035). Although no significance was indicated, CK20 showed a lower expression in the ≥ pT2 stage than in the pTa stage (57.1%–27.8%, *p* = 0.072). Regarding FGFR3 expression, the rate of high FGFR3 expression was maintained from the pTa stage to ≥pT2 (*p* = 0.822).

The K–M analysis revealed that the high CK5/6 and p53 expression groups showed significantly lower bladder cancer progression-free rates compared to their counterparts (*p* = 0.039 and *p* < 0.001, respectively; Figure 2d). Multivariate analysis indicated that high CK5/6 expression (HR = 4.56, *p* = 0.017) and high p53 expression (HR = 5.44, *p* = 0.002) were independent risk factors for MIBC progression, as well as the pT1 stage and high grade diagnosed at initial IVR (HR = 4.57, *p* = 0.044; HR = 1.95, *p* = 0.020, respectively; Table 4).

From the current results, CK20 high but CK5/6 low expression (luminal-like subtype) occupied the majority of IVR specimens in the initial case of bladder tumor recurrence. However, when bladder cancer subsequent to RNU progresses to muscle invasion status, the expression of CK20 and CK5/6 shifts to the opposite, suggesting a molecular transition to the basal-like subtype along with an increase in p53 expression (Figure 2e).

### 2.3. Biological Differences in IHC Staining of UTUC Metachronous and Bladder Primary MIBC

**Figure 3:**
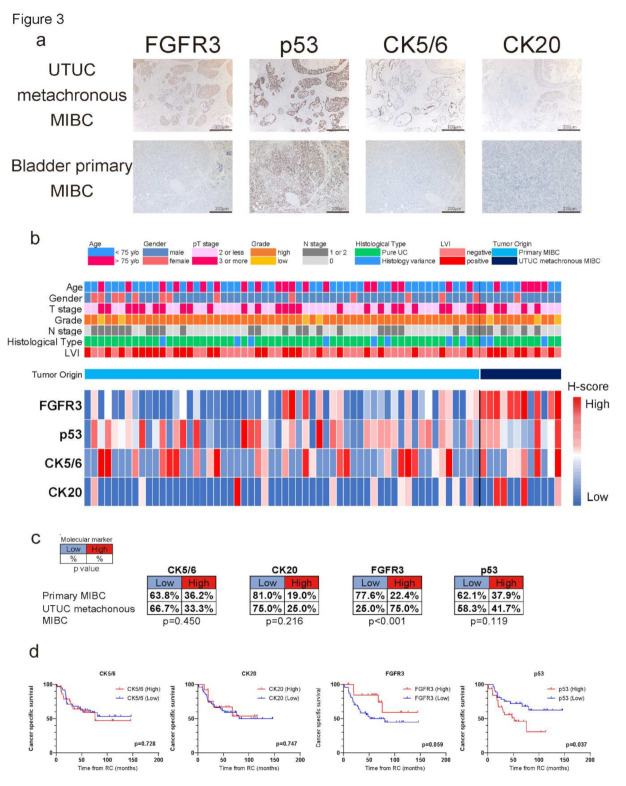
(**a**) Representative IHC staining of FGFR3, p53, CK5/6, and CK20 in surgical specimens after radical cystectomy (RC). pT2 UTUC metachronous MIBC and pT2 bladder primary MIBC are shown. Power field scale bar = 200 µm. (**b**) Heatmap describing the IHC score of each molecular marker in MIBC patients who underwent RC (*n* = 70). The heatmap of MIBC patients is classified by UTUC metachronous or bladder primary MIBC with information for age, sex, pT stage, grade, pN stage, histological type, and presence of LVI. (**c**) Chart describing the high/low expressions of all four molecular markers compared between UTUC metachronous and bladder primary MIBC. There were no significances in CK5/6, CK20, and p53 expressions between the two, while significantly higher FGFR3 expression was observed in UTUC metachronous MIBC compared to that of bladder primary MIBC. (**d**) K–M curves comparing the cancer-specific survival (CSS) rates in MIBC patients after RC compared by low versus high FGFR3 expression, low versus high p53 expression, low versus high CK5/6 expression, and low versus high CK20 expression. Log-rank test was used to assess the significance of differences. High p53 expression groups showed significantly lower CSS rates compared to the counterparts (*p* = 0.037). In contrast, high FGFR3 expression group showed higher CSS rates compared to the counterparts.

To confirm the biological differences between UTUC metachronous and bladder primary MIBC, we identified 12 UTUC metachronous and 58 bladder primary MIBC patients who underwent RC and conducted IHC staining of four molecular markers. The clinicopathological characteristics of the 70 patients with MIBC are shown in Table 5. The median age was 71 (43–90) and the median follow-up time was 47.2 (1.5–146.3) months. No significant differences were found between the two patient backgrounds. Overall, 32 (45.7%) patients died from MIBC. Representative IHC staining of UTUC metachronous and bladder primary MIBC is shown in Figure 3a. Biological differences were significant in the FGFR3 expression rate. Specifically, 9/12 (75%) of the patients showed high FGFR3 expression in UTUC metachronous MIBC, while 13 (22.4%) out of 58 patients showed high FGFR3 expression in bladder primary MIBC (*p* < 0.001; Figure 3b,c). K–M analysis indicated that the high FGFR3 expression group showed marginally significantly higher cancer-specific survival (CSS) rates than the low FGFR3 expression group (*p* = 0.059; Figure 3d). Multivariate analysis also showed that high FGFR3 expression and MIBC subsequent to UTUC were both favorable indicators of cancer-specific death (*p* = 0.051 and 0.002, respectively; Table 6).

### 2.4. Increase of Co-Occurrence Rate of FGFR3 and p53 Expression in UTUC Metachronous MIBC

**Figure 4:**
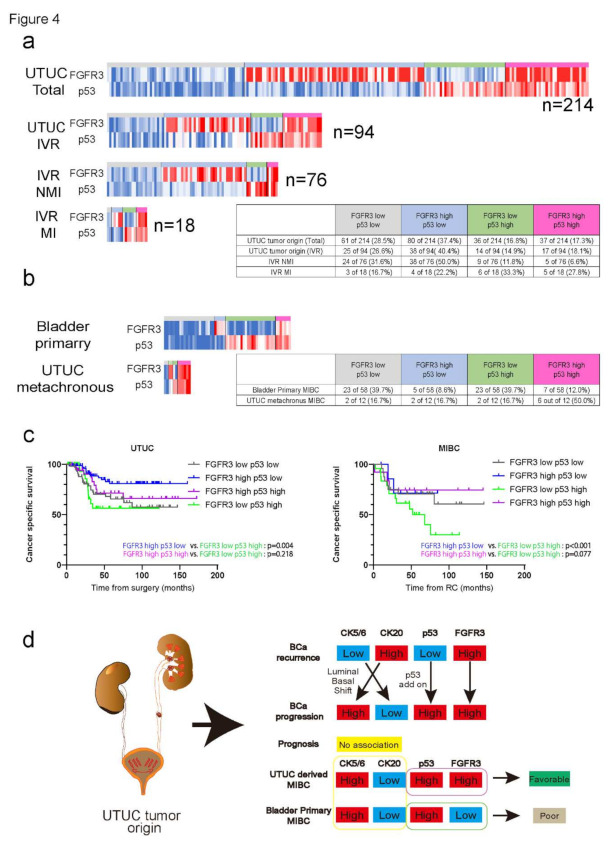
(**a**) Heatmap describing the relationships of FGFR3 and p53 expressions from UTUC tumor origin to MIBC progression. Gray bar shows both FGFR3 and p53 low group. Light blue bar shows FGFR3 high but p53 low group. Yellow-green bar shows FGFR3 low but p53 high group. Purple bar shows FGFR3 high p53 high group. Table chart shows the distributions of FGFR3 and p53 expression rates among UTUC tumor origin (*n* = 214), UTUC tumor origin limited to patients who developed IVR (*n* = 94), IVR tumor specimen (*n* = 94), and IVR tumor specimen that progressed to MIBC (*n* = 18). (**b**) Heatmap describing the relationships of FGFR3 and p53 expression in UTUC metachronous MIBC (*n* = 12) and bladder primary MIBC specimens (*n* = 58). Table chart shows the distributions of FGFR3 and p53 expression rates of the two. (**c**) K–M curves showing the CSS rates in UTUC patients after RNU (on the left) and MIBC patients after RC (on the right), classified by four groups: FGFR3 low/p53 low (gray line), FGFR3 high/p53 low (blue line), FGFR3 low/p53 high (green line), and FGFR3 high/p53 high (purple line). (**d**) Schema showing the entire biological characteristics and transitions of four molecular markers in UTUC patients. UTUC patients with luminal-like dependent features are likely to develop IVR, and the molecular characteristics are mostly taken over to IVR specimen. After bladder tumor recurrence, the molecular characteristics shift from luminal-like to basal-like features as the tumor progresses to muscle-invasive status. Although p53 and CK5/6 show higher expression in the MIBC stage, UTUC metachronous MIBC maintains higher FGFR3 expression compared with bladder primary MIBC, which may comprise favorable biological features after RC.

As we reviewed the molecular characteristics of the four biomarkers among UTUC tumor origin and IVR specimens, we observed that the co-occurrence of FGFR3 and p53 expression increased during the process of bladder cancer progression. We reconstructed the four markers’ IHC expression levels to match the UTUC and IVR specimens individually, as heatmap plots (Supplemental Figure S2). As shown in Figure 4, the rate of both the FGFR3 and p53 high expression groups gradually increased as it progressed to muscle-invasive status (UTUC tumor origin: 17.3% non-muscle-invasive IVR: 6.6%, and muscle-invasive IVR: 27.8%; Figure 4a). Regarding the comparison of UTUC metachronous and bladder primary MIBC, significantly higher rates of intermix of both FGFR3 and p53 expression were observed in UTUC metachronous MIBC (primary bladder MIBC: 12.0% vs. UTUC metachronous MIBC: 50.0%; Figure 4b). According to the K–M analysis, high FGFR3 with low p53 expression showed the most favorable survival outcomes in both UTUC and MIBC. Furthermore, both the FGFR3 and p53 high expression groups also demonstrated that they would still yield favorable outcomes in MIBC patients, while no significant differences were observed in UTUC patients (*p* = 0.077 and 0.218, respectively).

## 3. Discussion

In this study, we found that UTUC tumor origin, which comprises high CK20 and low CK5/6 expressions, is likely to develop IVR after RNU. By investigating the molecular expression in the IVR specimen, we also observed that p53 expression increased as the pT stage progressed to muscle-invasive status, and the majority of CK20 high and Ck5/6 low expressions would transition to CK20 low and CK5/6 high expressions (luminal–basal shift). Interestingly, UTUC metachronous MIBC revealed a different biological background compared to bladder primary MIBC, which comprises favorable biological features such as high FGFR3 expression rate (Figure 4d). To the best of our knowledge, this is the first study to report the molecular characteristics and transitions of UTUC tumor origin, IVR specimens, and bladder tumor specimens that progressed to MIBC.

Petros et al. demonstrated in their literature [7], from the sequential gene expression analysis of tumor samples from the same patient, that initial UTUC that develops IVR appears to be luminal-like, and metachronous bladder cancer after recurrence mainly takes over molecular characteristics such as luminal-like features. Since the literature was based on transcriptome analysis conducted by RNA sequences, our results also supported that UTUC tumor and IVR specimens share similar luminal-like phenotypes as confirmed by IHC staining, where FGFR3 and CK20 expressions occupy the majority of UTUC tumor origin and IVR specimens. In the process of repeated IVR development, however, we found that the CK20 high expression group gradually decreased, but the CK5/6 high expression group increased as the pT stage increased. The transition of CK5/6 and CK20 expression drastically changed when the bladder tumor progressed to muscle-invasive status. We suspect this phenomenon to be “luminal–basal shift” followed by an increase in p53 expression with MIBC progression. The concept of luminal subtype to basal shift has recently arisen. Tate et al. demonstrated a novel finding that the expression of an activated form of *P-parg* regulates bladder cancer subtype and immune exclusion [11]. In their experiment, they observed changes in the differentiation state of luminal tumors over time, suggesting a luminal to basal shift accompanied by downregulation of the *P-parg* gene. Regarding intra-tumor heterogeneity and high tumor mutational burden in UC, many possibilities may exist for determining the phenomenon of luminal–basal shift. Thus, further investigations and genomic experiments are required to clarify this question.

In this study, FGFR3 expression was maintained upon UTUC tumor origin, UTUC metachronous IVR, and UTUC metachronous MIBC progression (117/214 (54.7%) UTUC tumor origin, 52/94 (55.3%) IVR, and 9/18 (50.0%) UTUC metachronous MIBC). These expression rates are generally higher than those of the entire primary bladder MIBC, according to a recent study. Rhijin et al. have recently demonstrated that UC patients with FGFR3 mutations are significantly associated with longer disease-free survival, but patients with FGFR3 overexpression do not always guarantee favorable biological outcomes [12]. However, they still agree that FGFR3 overexpression is associated with lower pT stage and lower grade, and may be associated with favorable features, even though these subclones exhibit wild-type FGFR3 in bladder cancer. Since FGFR3 overexpression correlates with favorable outcomes in NMIBC and MIBC [13,14], we also revealed that UTUC metachronous MIBC may result in a favorable prognosis, which is consistent with the results of our previous report from a multi-institutional cohort study [15]. Although the clinical background of UTUC metachronous MIBC differs from that of primary bladder MIBC in terms of follow-up duration or frequency, total number of patients receiving perioperative chemotherapy, and low malignant potential of UTUC tumor origin, our molecular study provides one of the biological mechanisms for explaining the favorable outcomes of UTUC metachronous MIBC.

Finally, we found that the co-occurrence rate of both FGFR3 and p53 expression increased as UTUC tumor origin recurred to the bladder epithelium and progressed to MIBC. From the major genetic analyses conducted by recent studies [16,17], FGFR3 and p53 mutations are generally mutually exclusive. Therefore, co-occurrence of FGFR3 and p53 expression is relatively rare in a single tumor specimen. Although we could not perform mutational analysis in the entire tumor specimens, our results suggest that TP53 mutation may have occurred after FGFR3 mutation in the process of bladder recurrence or progression but maintained a favorable molecular biology even with the accumulation of gene mutations. However, one recent study reported that the co-occurrence of FGFR3 and mutant p53 expression was related to poor prognosis in lung carcinoma, which contrasts our molecular study [18]. Given the very small number and poor preservation status of tumor specimens for genomic analysis, the biological relationship and role between FGFR3 and p53 from UTUC tumor origin to metachronous MIBC deserves further investigation. Recently, the FGFR3 inhibitor erdafitinib has emerged as a new treatment agent for metastatic UC patients with FGFR3 alterations [19]. Perhaps FGFR3 expression confirmed by IHC staining could become the routine method for all UC patients; we expect that FGFR3 inhibitors can be considered for perioperative therapy and/or intravesical therapy for patients with UTUC who developed IVR or who progressed to MIBC afterwards since FGFR3 is constantly expressed in these tumor specimens.

This study had several limitations. The study population was relatively small and retrospectively examined. Not all of the patients had undergone complete regional lymphadenectomy, which may have led to the underestimation of the pathological staging of UTUC. Other molecular subtype markers, such as GATA3 and p63, were not used in this study. Although these biomarkers are representative IHC markers for nuclear staining and are known to provide more concrete and robust results, they were not deemed essential in our study. Since our objective was to compare the biological differences and transitions of primary UTUC tumors and IVR specimens, we had to limit our IHC markers to the four most common biomarkers for classifying UC subtypes. This study revealed the molecular characteristics of UTUC patients who developed IVR and progressed to MIBC only by IHC examination; therefore, no genomic analysis was conducted in our cohort. However, considering the rarity and long follow-up period of pursuing the tumor specimens from UTUC tumor origin to UTUC metachronous MIBC progression, we believe that it was extremely difficult to collect specimens with sufficient preservation for conducting accurate genomic analysis. All bladder tumors developed after RNU, and most bladder cancers can be classified as IVR from the upper tract. However, with respect to the 23 out of 65 IVR cases that had a previous history of bladder cancer, we could not rule out the possibility of some of the bladder tumors developing de novo in the urothelial epithelium [20].

## 4. Materials and Methods

### 4.1. Samples from UTUC Patients (Cohort 1)

This study was approved by the review board of Keio University Hospital (ID: 2013-0095). A total of 214 pTa-4N0-M0 UTUC patients who underwent RNU between 2000 and 2017 were identified and included in this study as Cohort 1. RNU was performed according to the standard procedure, involving extrafascial dissection of the kidney with the entire length of the ureter and adjacent segment of the bladder cuff being removed. A small iliac incision (Gibson incision) was made to retrieve the kidney and ureter en bloc and resect the bladder cuff. Regional lymph node dissection was not performed unless suspicious lymph nodes were detected on preoperative imaging or intraoperative findings.

### 4.2. Samples from NMIBC Tumor Specimens (Cohort 2)

Among the 214 patients with UTUC, 94 developed subsequent intravesical recurrence after radical nephrectomy. Intravesical recurrence was defined as the detection of a bladder tumor on cystoscopy and transurethral resection of the tumor.

The bladder tumor (TUR-BT) was then addressed according to the standard procedure. The use of intravesical therapy depended on the discretion of the attending physician. Bladder progression was defined as confirmed histologic muscle invasion in the bladder (pT2 or higher) during follow-up.

### 4.3. Samples from UTUC-Derived MIBC and Bladder Primary MIBC Tumor Specimens (Cohort 3)

To evaluate the molecular characteristics of UTUC-derived MIBC specimens, we identified 58 pT2-4N0-2M0 patients with bladder primary MIBC who underwent radical cystectomy (RC) between 2006 and 2016 at the same institutions and compared the molecular marker expression with UTUC metachronous MIBC. All patients underwent standard RC using an open surgical method with urinary diversion, including an ileal conduit, neobladder, or cutaneous ureterostomy. Regional lymphadenectomy, including the bilateral internal iliac, external iliac, and obturator lymph nodes, was performed.

### 4.4. Evaluation of UC Specimen after Surgical Management

Surgical specimens were processed according to the standard pathological procedures. All specimens, including primary UTUC, subsequent IVR, and MIBC, were histologically confirmed to be urothelial carcinomas. Tumors were staged according to the 2002 American Joint Committee on Cancer/International Union for Cancer Control TNM classification and graded according to the 2004 World Health Organization classification [21]. Lymphovascular invasion (LVI) was defined as the unequivocal presence of tumor cells within endothelial-lined lymphatic and vascular channels based on the criteria in the “WHO Classification of Tumours of the Urinary System and Male Genital Organs [22].” Tumor multifocality was defined as the presence of pathologically confirmed tumors in at least two distinct locations within the upper urinary tract that involve the renal pelvis and ureter [23].

### 4.5. Follow-Up Regimen

**Patients with UTUC** were followed up routinely 3–6 months after surgery, every 6 months during the first 5 years and annually thereafter. The follow-up consisted of a history, physical examination, routine blood work, urinary cytology, chest radiography, and cystoscopy of the urinary bladder. Radiographic evaluations of the contralateral upper urinary tract using computed tomography (CT), magnetic resonance imaging, or excretory urography were conducted every 6 months for the first 5 years and annually thereafter. Elective bone scans and chest CT were performed when clinically indicated.

Patients with MIBC received RC and were generally followed up at least every 3 to 4 months for 2 years, then every 6 months until 5 years, and annually thereafter. Radiographic evaluations were conducted using the same flow as those for UTUC. Bladder recurrence-free survival was calculated as the duration from RNU to the date when bladder tumor recurrence was detected. Cancer-specific survival (CSS) was defined as the period from RNU or RC to cancer-related deaths from urothelial carcinoma.

### 4.6. Tissue Samples and Immunohistochemical Examination

We examined 214 UTUC tumor specimens, including 94 IVR tumor specimens (specimens from 76 NMIBC cases and 18 MIBC cases) that subsequently developed after RNU. Regarding RC specimens, 12 RC specimens of UTUC metachronous MIBC and another 58 specimens from bladder primary MIBC patients were obtained. IHC staining was performed using the unstained sliced slide taken from one key paraffin block per case. We used four representative biomarkers: FGFR3, p53, CK5/6, and CK20. Specimens were fixed in 10% formalin and embedded in paraffin, and all slides were reviewed again by genitourinary pathologists. Sections (4 µm thickness) of formalin-fixed and paraffin-embedded tissues were evaluated. Sections were deparaffinized in xylene and rehydrated in graded alcohol and distilled water. After antigen retrieval with citric acid (pH 6.0) at 120 °C for 10 min, endogenous peroxidase activity was blocked with 1% hydrogen peroxide for 15 min, followed by washing with distilled water. To bind nonspecific antigens, sections were incubated at room temperature for 15 min with 5% skim milk in phosphate-buffered saline (PBS). The sections were then incubated at 4 °C overnight with anti-FGFR3 mouse monoclonal antibody (Ab; 1:150 dilution; Origene, Rockville, MD, USA), anti-p53 mouse monoclonal Ab (1:2000 dilution; Dako, Santa Clara, CA, USA), anti-CK5/6 mouse monoclonal Ab (1:100, Invitrogen, Waltham, MA, USA), and anti-CK20 mouse monoclonal Ab (1:100, Abcam, Cambridge, UK). After washing with PBS, the tissue sections were incubated with the secondary Ab for 60 min. Color was developed using 3,30-diaminobenzidine tetrahydrochloride in 50 mmol/L Tris-HCl (pH 7.5) containing 0.005% hydrogen peroxide. The sections were then counterstained with hematoxylin.

To assess IHC staining, cancer cells with positive staining in the cell cytoplasm or nucleus were counted in at least 10 representative fields, and the mean percentage of positive cancer cells (0–100) and staining intensity stratified from 0 to 3 (0: no staining, 1: low staining, 2: moderate staining, 3: strong staining) were estimated [24,25]. The histoscore (H-score) was calculated using the following formula: mean percentage × intensity (range 0-300) [26].

### 4.7. Statistical Analysis

Medians and interquartile ranges were generated for continuously coded variables, and the Mann–Whitney *U* and chi-square tests were used to assess the significance of differences between medians and proportions, respectively. A one-way ANOVA was conducted to compare three or more continuous variables. Kaplan–Meier (K–M) analyses with log-rank tests were conducted to draw the RFS and CSS curves. Univariate and multivariate Cox regression models were used to calculate proportional hazard ratios (HRs) to investigate the risk factors for MIBC progression and prognostic indicators for MIBC patients. In all statistical analyses, tests were two-sided, and statistical significance was set at a *p* value < 0.05. All statistical analyses were performed using the GraphPad Prism 8 software and Statistical Package of Social Sciences software (version 24.0; SPSS, Chicago, Illinois, USA).

## 5. Conclusions

Overall, we conducted a molecular analysis to investigate the biological features of UTUC tumor origin, subsequent bladder tumor recurrence, and bladder tumor progression. We found that UTUC tumor origin and subsequent bladder recurrence comprised similar molecular characteristics, such as luminal-like subtypes. In the process of MIBC progression, the luminal-like characteristics transition to basal-like characteristics as NMIBC progresses to MIBC. Since UTUC metachronous MIBC has higher FGFR3 expression compared with primary bladder MIBC, it suggests that MIBC following UTUC may take over FGFR3 expression from UTUC tumor origin, which may have resulted in favorable survival outcomes with additional RC compared to that of bladder primary MIBC.

## Figures and Tables

**Table 1:** Clinicopathological and molecular characteristics of UTUC patients who underwent radical nephroureterectomy.

	Patients Who Underwent RNU	Total	Patients with IVR	Patients without IVR	*p* Value
Patient Characteristics		*n* = 214	*n* = 94, (%)	*n* = 120, (%)	
Age	<75	107 (50.0)	53 (49.5)	54 (50.5)	0.065
	≥75	107 (50.0)	41 (38.3)	66 (61.7)	
Sex	male	158 (73.8)	77 (48.7)	81 (51.3)	0.012
	female	56 (26.2)	17 (30.4)	39 (69.6)	
Tumor location	pelvis	118 (55.1)	50 (42.4)	68 (57.6)	0.356
	ureter	96 (44.9)	44 (46.8)	52 (53.2)	
Tumor histology	pure UC	171 (79.9)	80 (46.8)	91 (53.2)	0.065
	UC with histology variants	43 (20.1)	14 (32.6)	29 (67.4)	
Pathological T stage	≤ 2	116 (54.2)	64 (55.2)	52 (44.8)	<0.001
	≥ 3	98 (45.8)	30 (30.6)	68 (69.4)	
Pathological N stage	0	49 (22.9)	23 (46.9)	26 (53.1)	0.099
	1,2	15 (7.0)	2 (13.3)	13 (86.7)	
	Nx	150 (70.1)	69 (46.0)	81 (54.0)	
Lymph node dissection	no	150 (70.1)	69 (46.0)	81 (54.0)	0.216
	yes	64 (29.9)	25 (39.1)	39 (60.9)	
Surgical margin	negative	189 (88.3)	87 (46.0)	102 (54.0)	0.066
	positive	25 (11.7)	7 (28.0)	18 (72.0)	
Tumor grade	low	64 (29.9)	36 (56.3)	28 (43.7)	0.013
	high	150 (70.1)	58 (38.7)	92 (61.3)	
LVI	absent	118 (55.1)	62 (52.5)	56 (47.5)	0.004
	present	96 (44.9)	32 (33.3)	64 (66.7)	
Concomitant CIS	no	170 (79.4)	74 (43.5)	96 (56.5)	0.475
	yes	44 (20.6)	20 (45.4)	24 (54.6)	
Tumor multifocality	no	182 (85.0)	79 (43.4)	103 (56.6)	0.430
	yes	32 (15.0)	15 (46.9)	17 (53.1)	
Systemic adjuvant chemotherapy	no	140 (65.4)	65 (46.4)	75 (53.6)	0.192
	yes	74 (34.6)	29 (39.2)	45 (60.8)	
Previous history of bladder cancer	no	149 (69.6)	71 (47.7)	78 (52.3)	0.065
	yes	65 (30.4)	23 (35.4)	42 (64.6)	
Exrtravesical tumor recurrence	no	146 (68.2)	68 (46.6)	78 (53.4)	0.159
	yes	68 (31.8)	26 (38.2)	42 (61.8)	
Cancer specific Death	no	162 (75.7)	73 (45.1)	89 (54.9)	0.334
	yes	52 (24.3)	21 (40.4)	31 (59.6)	

Abbreviations: UTUC; upper tract urothelial carcinoma, IVR; intravesical recurrence, SD; standard deviation, UC; urothelial carcinoma, LVI; lymphovascular invasion, CIS; carcinoma in situ.

**Table 2:** Uni- and multivariate Cox regression analyses for determining risk factors for IVR development in UTUC patients. Abbreviations: IVR; intravesical recurrence, UTUC; upper tract urothelial carcinoma, HR; hazard ratio, CI; confidence interval, UC; urothelial carcinoma, CIS; carcinoma in situ, LVI; lymphovascular invasion, FGFR; fibroblast growth factor receptor, CK; cytokeratin.

	Univariate			Multivariate		
Clinical Indicators	HR	95% CI	*p* Value	HR	95% CI	*p* Value
Age (≥ 75 vs. <75)	0.69	0.45–1.06	0.086			
Sex (male vs. female)	0.64	0.37–1.11	0.113			
Tumor location (ureter vs. renal pelvis)	1.17	0.75–1.83	0.500			
Tumor histology (pure UC vs. non-pure UC)	1.23	0.61–2.46	0.564			
Pathological T stage (≤T2 vs. ≥T3)	1.93	1.15–3.22	0.013	1.94	1.20–3.15	0.007
Pathological N stage (N0 vs. N1,2)	0.65	0.14–3.11	0.590			
Tumor grade (high vs. low)	1.56	0.89–2.75	0.121	1.61	1.04–2.49	0.032
Concomitant CIS (yes vs. no)	1.02	0.53–1.98	0.955			
Tumor multifocality (yes vs. no)	1.38	0.69–2.77	0.365			
LVI (positive vs. negative)	0.74	0.41–1.35	0.326			
Systemic adjuvant chemotherapy (yes vs. no)	1.21	0.74–2.00	0.449			
Previous history of bladder tumor (yes vs. no)	0.58	0.70–1.90	0.581			
FGFR3 expression (high vs. low)	1.34	0.85–2.13	0.210			
p53 expression (high vs. low)	0.74	0.42–1.29	0.287			
CK5/6 expression (high vs. low)	0.29	0.144–0.590	0.001	0.26	0.14–0.49	<0.001
CK20 expression (high vs. low)	3.17	1.91–5.26	<0.001	3.31	2.10–5.23	<0.001

Abbreviations: IVR; intravesical recurrence, UTUC; upper tract urothelial carcinoma, HR; hazard ratio, CI; confidence interval, UC; urothelial carcinoma, CIS; carcinoma in situ, LVI; lymphovascular invasion, FGFR; fibroblast growth factor receptor, CK; cytokeratin.

**Table 3:** Clinicopathological characteristics of UTUC patients who developed IVR. Abbreviations: UTUC; upper tract urothelial carcinoma, IVR; intravesical recurrence, MIBC; muscle invasive bladder cancer, UC; urothelial carcinoma, LVI; lymphovascular invasion, CIS; carcinoma in situ, BT: bladder tumor.

		Total	MIBC Progression	non-MIBC Progression	*p* Value
Patient Characteristics		*n* = 94, (%)	*n* = 18, (%)	*n* = 76, (%)	
Age	<75	41 (43.6)	10 (24.4)	31 (75.6)	0.477
	≥75	53 (56.4)	8 (15.1)	45 (84.9)	
Sex	male	77 (81.9)	12 (15.6)	65 (84.4)	0.143
	female	17 (18.1)	6 (35.3)	11 (64.7)	
Pathological T stage (Initial IVR)	a	37 (39.4)	2 (5.4)	35 (94.6)	0.005
	1	51 (54.3)	10 (19.6)	41 (80.4)	
	2	6 (6.3)	6 (100.0)	0 (0.0)	
Tumor grade (Initial IVR)	low	48 (51.1)	5 (10.4)	43 (89.6)	0.026
	high	46 (48.9)	13 (28.3)	33 (71.7)	
LVI (Initial IVR)	absent	73 (77.7)	8 (11.0)	65 (89.0)	0.001
	present	21 (22.3)	10 (47.6)	11 (52.4)	
Concomitant CIS (Initial IVR)	no	83 (88.3)	15 (18.1)	68 (81.9)	0.352
	yes	11 (11.7)	3 (27.3)	8 (72.7)	
Recurrence times of BT	1	35 (37.2)	6 (17.1)	29 (82.9)	<0.001
	2	21 (22.3)	5 (23.8)	16 (76.2)	
	3	12 (12.8)	3 (25.0)	9 (75.0)	
	4	24 (25.5)	4 (16.7)	20 (83.3)	
	5	2 (2.1)	0 (0.0)	2 (100.0)	
Intravesical chemotherapy	no	19 (20.2)	6 (31.6)	13 (68.4)	0.115
	yes	75 (79.8)	12 (16.0)	63 (84.0)	

Abbreviations: UTUC; upper tract urothelial carcinoma, IVR; intravesical recurrence, MIBC; muscle invasive bladder cancer, UC; urothelial carcinoma, LVI; lymphovascular invasion, CIS; carcinoma in situ, BT: bladder tumor.

**Table 4:** Uni- and multivariate Cox regression analyses for determining risk factors for MIBC progression after IVR development. Abbreviations: MIBC; muscle invasive bladder cancer, IVR; intravesical recurrence, HR; hazard ratio, CI; confidence interval, UC; urothelial carcinoma, CIS; carcinoma in situ, BT: bladder tumor, FGFR; fibroblast growth factor receptor, CK; cytokeratin.

	Univariate			Multivariate		
Clinical Indicators	HR	95% CI	*p* Value	HR	95% CI	*p* Value
Age (≥75 vs. <75)	0.71	0.17–2.93	0.636			
Sex (male vs. female)	1.19	0.29–4.83	0.807			
Pathological T stage (T1 vs. Ta)	6.62	1.04-24.0	0.045	4.57	1.04–20.0	0.044
Tumor grade (high vs. low)	2.63	1.44–4.78	0.020	1.95	1.27–3.00	0.020
Concomitant CIS (yes vs. no)	1.27	0.20–8.00	0.802			
Intravesical therapy (yes vs. no)	0.69	0.18–2.68	0.591			
Recurrence times of BT (3 or more vs. 2 or low)	1.47	0.36–6.10	0.591			
FGFR3 expression (high vs. low)	0.56	0.12–4.07	0.674			
p53 expression (high vs. low)	4.81	1.46–15.8	0.010	5.44	1.85–16.0	0.002
CK5/6 expression (high vs. low)	5.96	1.27–28.0	0.024	4.56	1.31–15.8	0.017
CK20 expression (high vs. low)	0.68	0.11–4.07	0.674			

Abbreviations: MIBC; muscle invasive bladder cancer, IVR; intravesical recurrence, HR; hazard ratio, CI; confidence interval, UC; urothelial carcinoma, CIS; carcinoma in situ, BT: bladder tumor, FGFR; fibroblast growth factor receptor, CK; cytokeratin.

**Table 5:** Patient and tumor characteristics of MIBC patients who underwent radical cystectomy. Abbreviations: MIBC; muscle invasive bladder cancer, UTUC; upper tract urothelial carcinoma, LVI; lymphovascular invasion.

		Total	UTUC Derived MIBC	Bladder Primary MIBC	
Patient Characteristics		*n* = 70, (%)	*n* = 12, (%)	*n* = 58, (%)	*p* Value
Age	<75	45 (64.3)	7 (58.3)	38 (65.5)	0.744
	≥75	25 (35.7)	5 (41.7)	20 (34.5)	
Sex	male	56 (80.0)	11 (91.7)	45 (77.6)	0.248
	female	14 (20.0)	1 (8.3)	13 (22.4)	
Pathological T stage	<3	39 (55.7)	7 (58.3)	32 (55.2)	0.509
	3 or more	31 (44.3)	5 (41.7)	26 (44.8)	
Pathological N stage	0	41 (58.6)	6 (50.0)	35 (60.3)	0.560
	1,2	29 (41.4)	6 (50.0)	23 (39.7)	
Tumor grade	low	7 (10.0)	3 (25.0)	4 (6.9)	0.092
	high	63 (90.0)	9 (75.0)	54 (93.1)	
LVI	absent	38 (54.3)	5 (41.7)	33 (56.9)	0.259
	present	32 (45.7)	7 (58.3)	25 (43.1)	
Systemic chemotherapy	no	19 (27.2)	4 (33.3)	15 (25.9)	0.491
	yes	51 (72.8)	8 (66.7)	43 (74.1)	
Cancer death	no	38 (54.3)	10 (83.3)	28 (48.3)	0.002
	yes	32 (45.7)	2 (16.7)	30 (51.7)	

Abbreviations: MIBC; muscle invasive bladder cancer, UTUC; upper tract urothelial carcinoma, LVI; lymphovascular invasion.

**Table 6:** Uni- and multivariate analyses evaluating the prognostic factors associated with oncological outcomes in MIBC patients (*n* = 70). Abbreviations: MIBC; muscle-invasive bladder cancer, HR; hazard ratio, CI; confidence interval, UTUC; upper tract urothelial carcinoma, LVI; lymphovascular invasion, FGFR; fibroblast growth factor receptor, CK; cytokeratin.

	Disease Recurrence						Cancer-Specific Death					
	Univariate			Multivariate			Univariate			Multivariate		
Clinical Indicators	HR	95% CI	*p* value	HR	95% CI	*p* Value	HR	95% CI	*p* Value	HR	95% CI	*p* value
Age (≥75 vs. <75)	1.62	0.66–3.99	0.298				1.25	0.45–3.49	0.667			
Sex (male vs. female)	1.26	0.51–3.11	0.614				1.04	0.43–2.52	0.924			
Systemic chemotherapy (yes vs. no)	0.38	0.12–0.91	0.043	0.24	0.111–0.497	0.001	1.20	0.47–3.09	0.699			
Pathological T stage (≥3 vs. <3)	2.59	0.97–6.94	0.059	2.47	1.05–5.76	0.037	2.95	1.15–7.55	0.024	2.40	1.14-5.06	0.021
Pathological N stage (1,2 vs. 0)	3.11	1.22–7.97	0.614	2.86	1.40–5.84	0.004	4.18	1.62–10.8	0.003	3.07	1.27-7.41	0.013
UTUC metachronous MIBC (yes vs. no)	0.56	0.11–2.96	0.493				0.39	0.12–1.21	0.103	0.14	0.02-0.88	0.002
Tumor grade (high vs. low)	2.93	0.32–2.63	0.338				2.09	0.52–8.54	0.301			
LVI (positive vs. negative)	2.50	0.88–5.52	0.084				2.73	1.00–7.45	0.050			
FGFR3 (high vs. low)	0.35	0.12–0.94	0.013	0.32	0.13–0.79	0.013	0.47	0.20–1.10	0.082	0.29	0.02-1.01	0.051
p53 (high vs. low)	1.78	0.75–4.25	0.194				4.29	1.73–10.6	0.002	2.56	0.131-0.623	0.008
CK5/6 (high vs.low)	1.47	0.64–3.03	0.371				2.15	0.94–4.89	0.070			
CK20 (high vs.low)	0.92	0.31–2.68	0.873				1.07	0.44–2.64	0.880			

Abbreviations: MIBC; muscle-invasive bladder cancer, HR; hazard ratio, CI; confidence interval, UTUC; upper tract urothelial carcinoma, LVI; lymphovascular invasion, FGFR; fibroblast growth factor receptor, CK; cytokeratin.

## Data Availability

Not applicable.

## Supplementary Materials

The following supporting information can be downloaded at: https://www.mdpi.com/article/10.3390/ijms23095154/s1.
