# Supplementary figures and images for: Profiling the Biological Characteristics and Transitions through Upper Tract Tumor Origin, Bladder Recurrence, and Muscle-Invasive Bladder Progression in Upper Tract Urothelial Carcinoma
Source: Int J Mol Sci. 2022 May 5;23(9):5154. doi: 10.3390/ijms23095154 (PMC9105227; doi:10.3390/ijms23095154)

Supplemental Figure S1

a: n=214

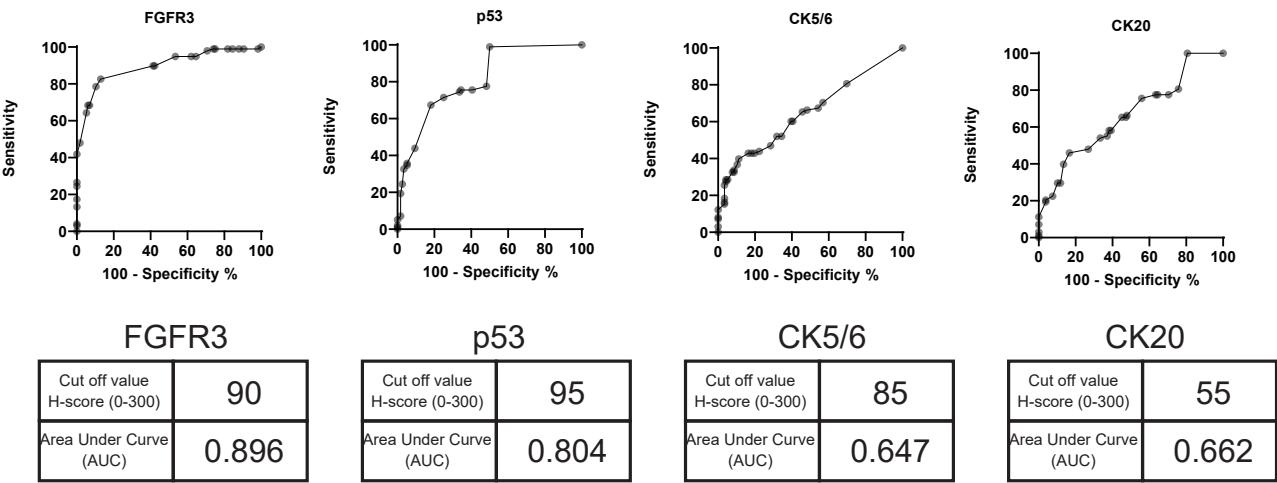

b: n=94

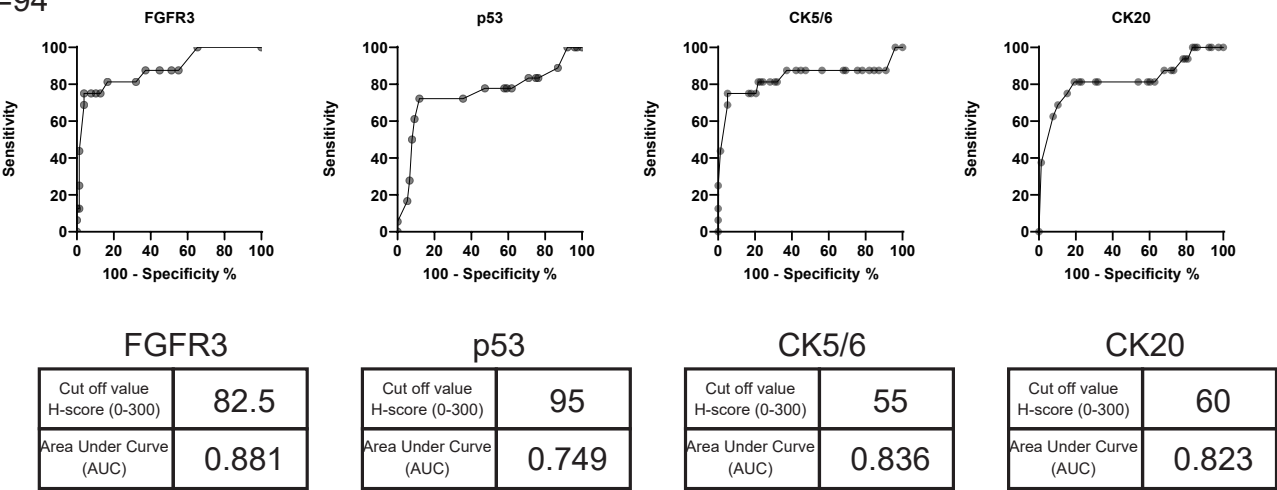

c: n=70

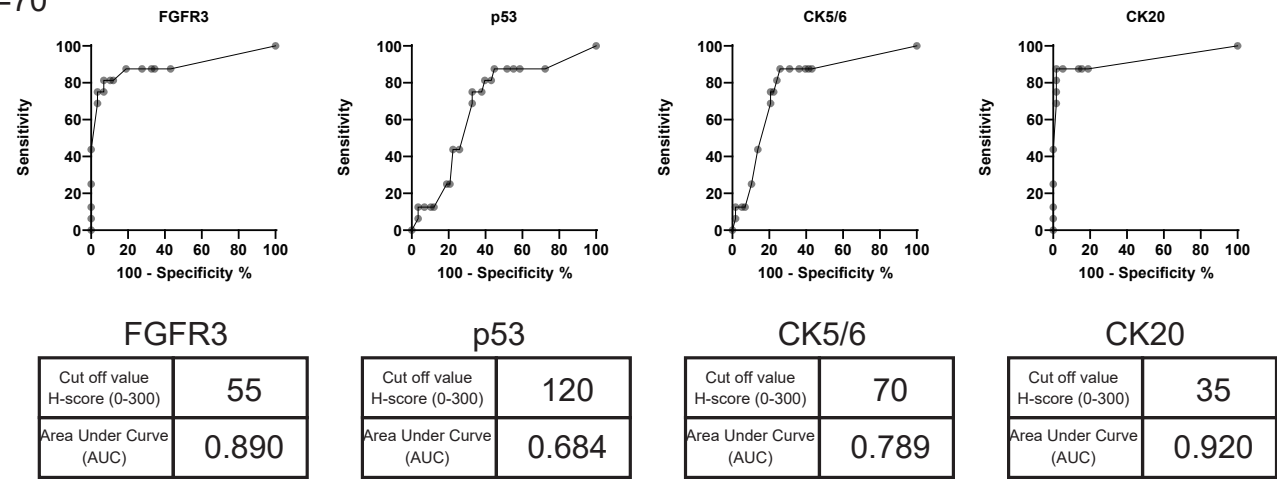

a FGFR3

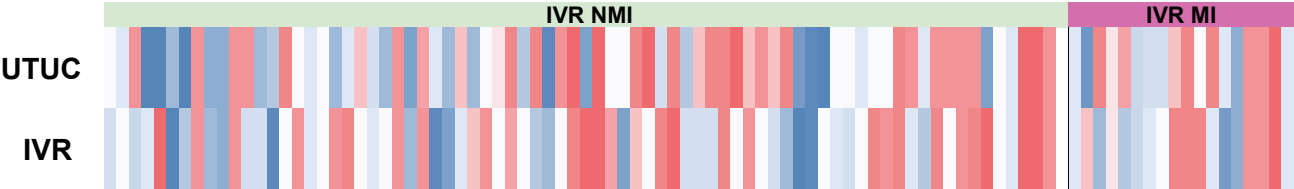

b p53

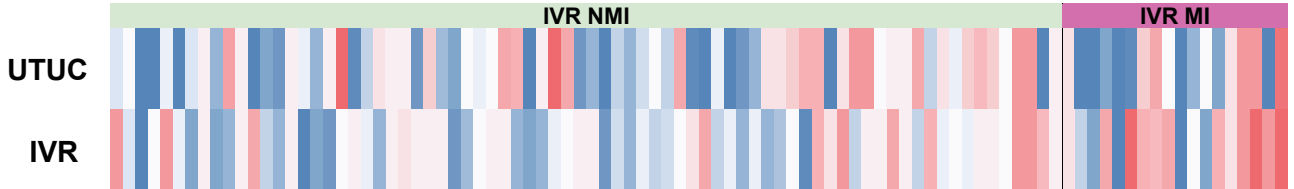

c CK5/6

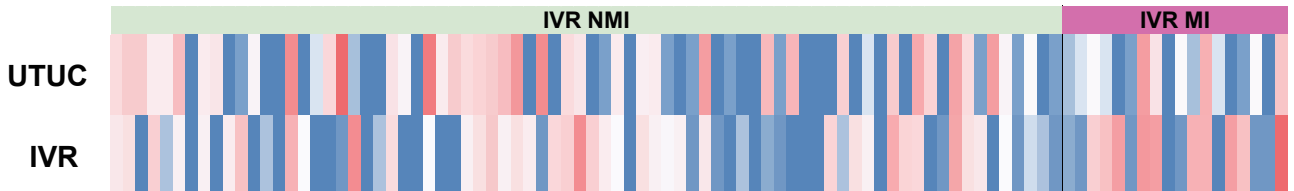

d CK20

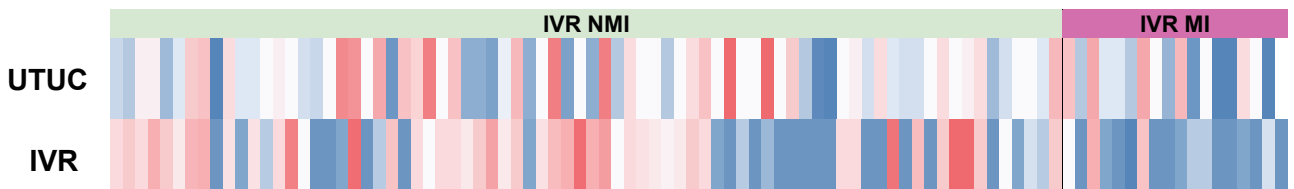

Supplement: Supplementary file 1 [file ijms-23-05154-s001.zip › ijms-1672693-supplementary.pdf]
